# Supplementary figures and images for: Survey of the Heritability and Sparse Architecture of Gene Expression Traits across Human Tissues
Source: PLoS Genet. 2016 Nov 11;12(11):e1006423. doi: 10.1371/journal.pgen.1006423 (PMC5106030; doi:10.1371/journal.pgen.1006423)

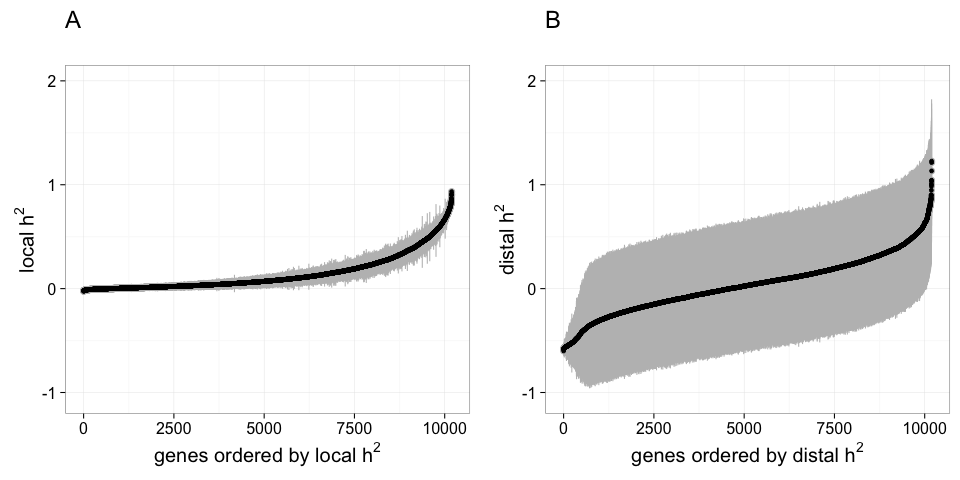

Supplement: S1 Fig — In order to obtain an unbiased estimate of mean h2, we allow the values to be negative when fitting the restricted maximum likelihood (unconstrained REML). (A) Local (SNPs within 1Mb of each gene) and (B) distal (all SNPs on non-gene chromosomes) gene expression h2 estimates ordered by increasing h2. Gray segments show two times the standard errors of each h2 estimate. Notice the larger range and errors of the distal estimates. (PNG) [file pgen.1006423.s001.png]

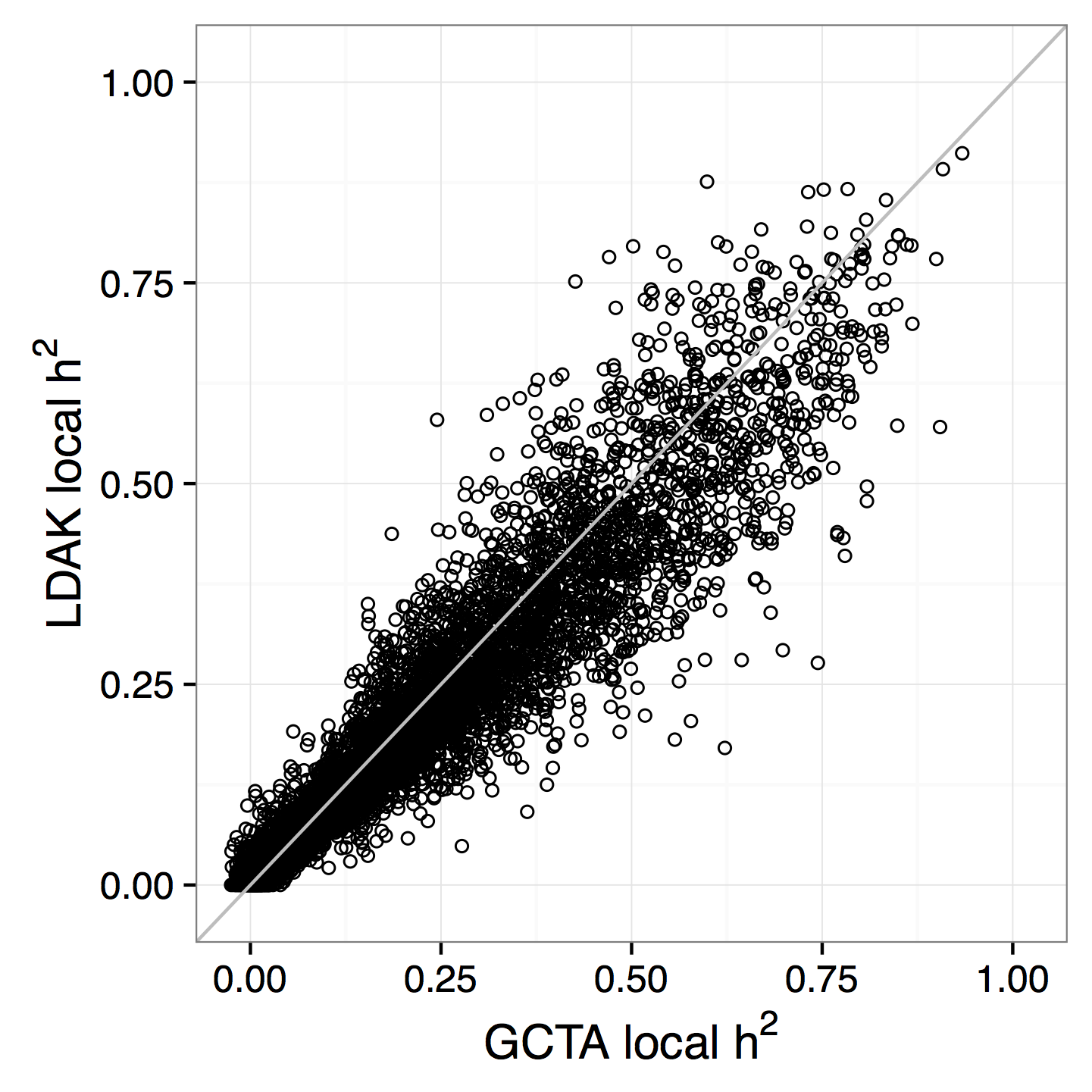

Supplement: S2 Fig — The Pearson correlation between GCTA and the LD-adjusted kinship local h2 estimates of gene expression is 0.96. (PNG) [file pgen.1006423.s002.png]

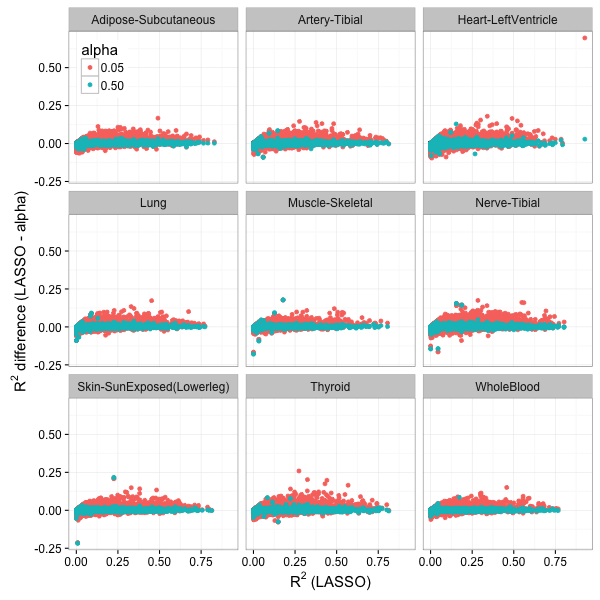

Supplement: S3 Fig — The difference between the cross validated R2 of the LASSO model and the elastic net model mixing parameters 0.05 and 0.5 for autosomal protein coding genes per tissue. Elastic net with α = 0.5 values hover around zero, meaning that it has similar predictive performance to LASSO. The R2 difference of the more polygenic model (elastic net with α = 0.05) is mostly above the 0 line, indicating that this model performs worse than the LASSO model across tissues. (PNG) [file pgen.1006423.s003.png]

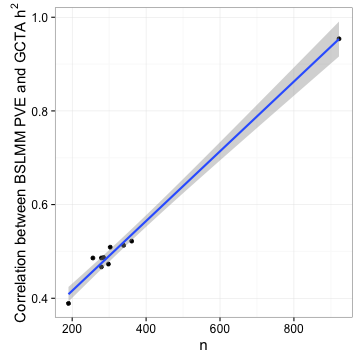

Supplement: S4 Fig — The Pearson correlation between estimates of heritability using BSLMM vs. GCTA is dependent on sample size (R = 0.99, P = 3x10^−9^). (PNG) [file pgen.1006423.s004.png]

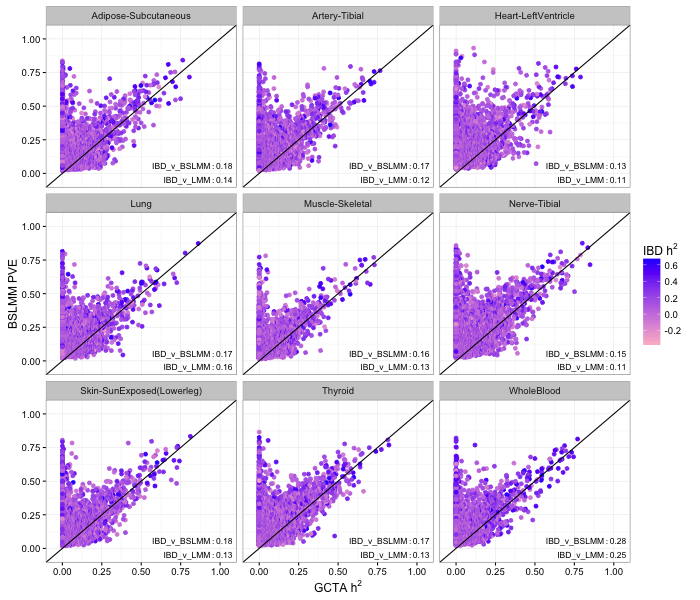

Supplement: S5 Fig — This figure shows the comparison between estimates of heritability using BSLMM vs. LMM (GCTA) for GTEx data. Here, in both models the estimates are constrained to be between 0 and 1. For most genes BSLMM estimates are larger than LMM estimates reflecting the fact that BSLMM yields better estimates of heritability because of its ability to account for the sparse component. Each point is colored according to that gene’s estimate of h2 by shared identity by descent (IBD) in Price et al. [29]. At the bottom right of each panel, we show the IBD correlation with BSLMM (IBD_v_BSLMM) and LMM (IBD_v_LMM). BSLMM is consistently more correlated with the IBD estimate. This provides further evidence that LMM is underestimating h2 for these genes. (PNG) [file pgen.1006423.s005.png]

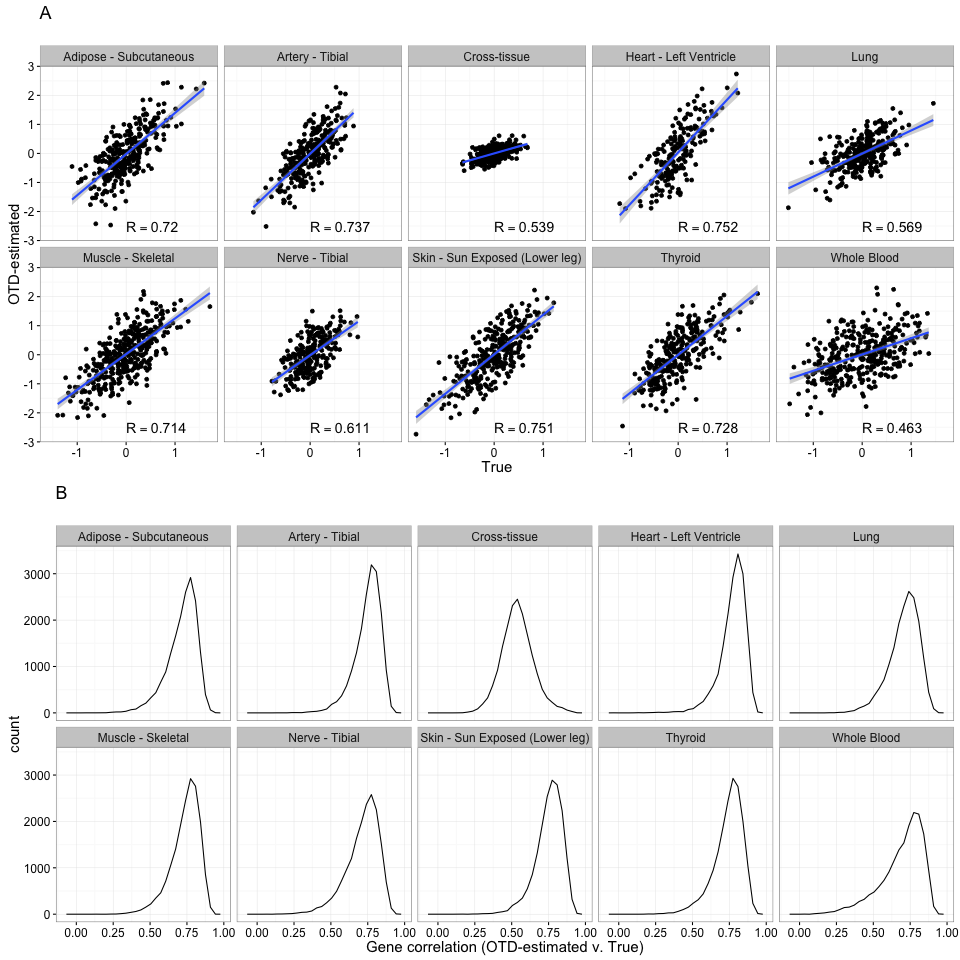

Supplement: S6 Fig — The simulated phenotype is the sum of the observed cross-tissue and tissue-specific components (estimated via OTD in the observed data) plus an error term derived by randomly sampling a normal distribution with variance equal to the variance of each gene. (A) Correlation between true and OTD-estimated cross-tissue and tissue-specific components for one gene, SLTM. (B) Histogram of the correlation between true and OTD-estimated components across all genes. (PNG) [file pgen.1006423.s006.png]

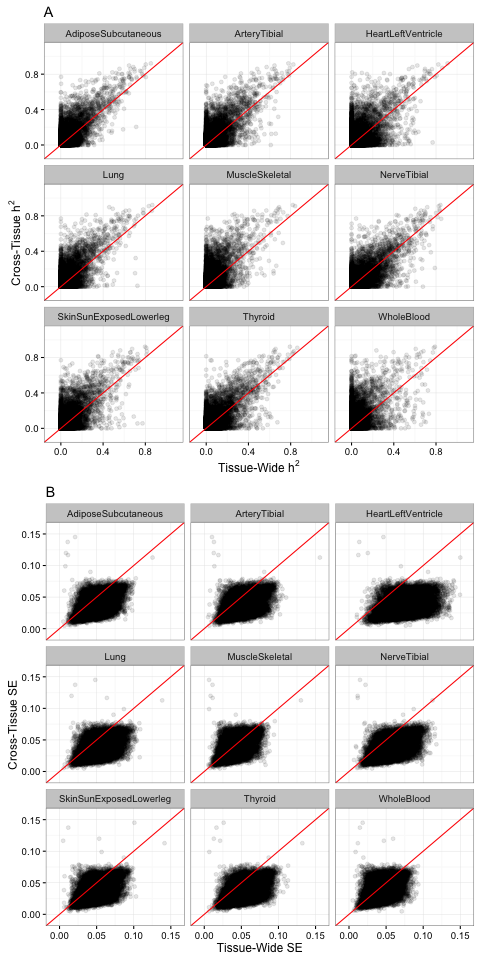

Supplement: S7 Fig — Cross-tissue local h2 is estimated using the cross-tissue component (random effects) of the mixed effects model for gene expression and SNPs within 1 Mb of each gene. Whole tissue local h2 is estimated using the measured gene expression for each respective tissue and SNPs within 1 Mb of each gene. Estimates of h2 for cross-tissue expression traits are larger and their standard errors are smaller than the corresponding estimates for each whole tissue expression trait. (PNG) [file pgen.1006423.s007.png]

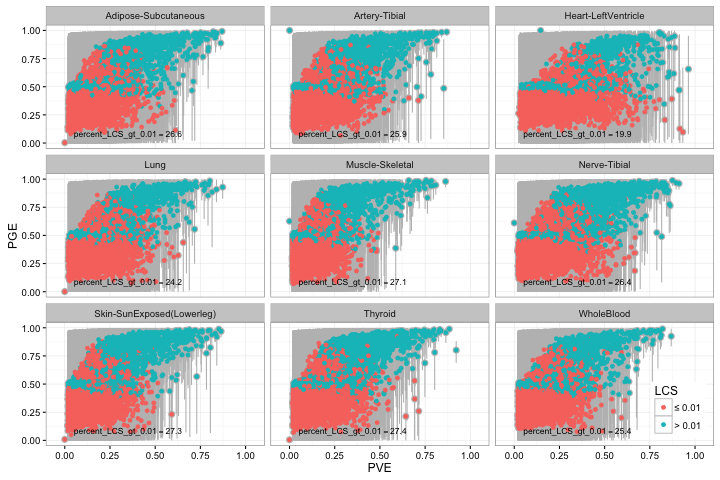

Supplement: S8 Fig — Comparison of median PGE (proportion of PVE explained by sparse effects) to median PVE (total proportion of variance explained, the BSLMM equivalent of h2) for expression of each gene. The 95% credible set of each PGE estimate is in gray and genes with a lower credible set (LCS) greater than 0.01 are in blue. For highly heritable genes the sparse component is close to 1, thus for high heritability genes the local architecture is sparse across tissues. For lower heritability genes, there is not enough evidence to determine sparsity or polygenicity. (PNG) [file pgen.1006423.s008.png]

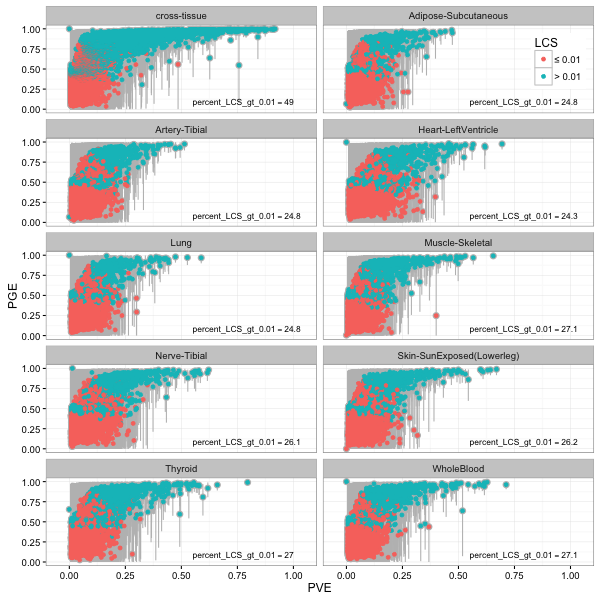

Supplement: S9 Fig — Comparison of median PGE (proportion of PVE explained by sparse effects) to median PVE (total proportion of variance explained, the BSLMM equivalent of h2) for expression of each gene. The 95% credible set of each PGE estimate is in gray and genes with a lower credible set (LCS) greater than 0.01 are in blue. For highly heritable genes the sparse component is close to 1, thus for high heritability genes the local architecture is sparse across tissues. About twice as many cross-tissue expression traits have significant PGE (LCS > 0.01) compared to the tissue-specific expression traits. (PNG) [file pgen.1006423.s009.png]

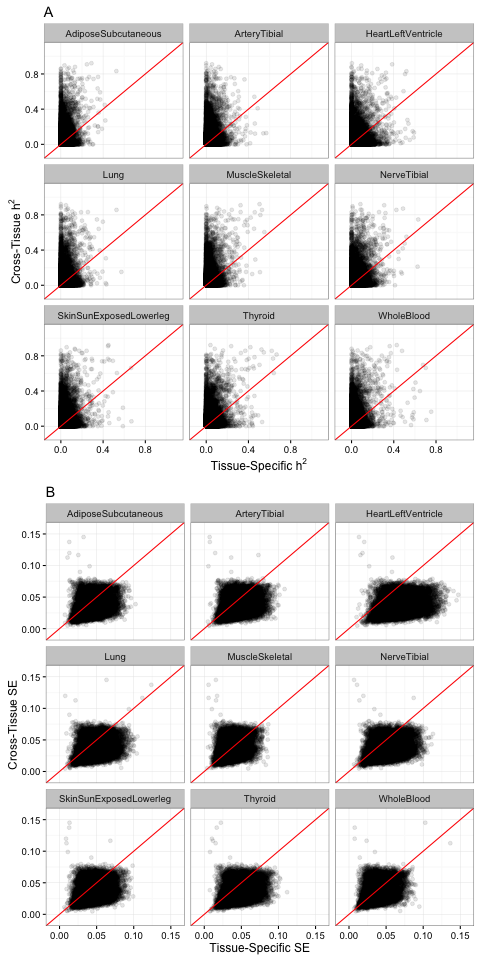

Supplement: S10 Fig — Cross-tissue local h2 is estimated using the cross-tissue component (random effects) of the mixed effects model for gene expression and SNPs within 1 Mb of each gene. Tissue-specifc local h2 is estimated using the tissue-specific component (residuals) of the mixed effects model for gene expression for each respective tissue and SNPs within 1 Mb of each gene. Estimates of h2 for cross-tissue expression traits are larger and their standard errors are smaller than the corresponding estimates for each tissue-specific expression trait. (PNG) [file pgen.1006423.s010.png]

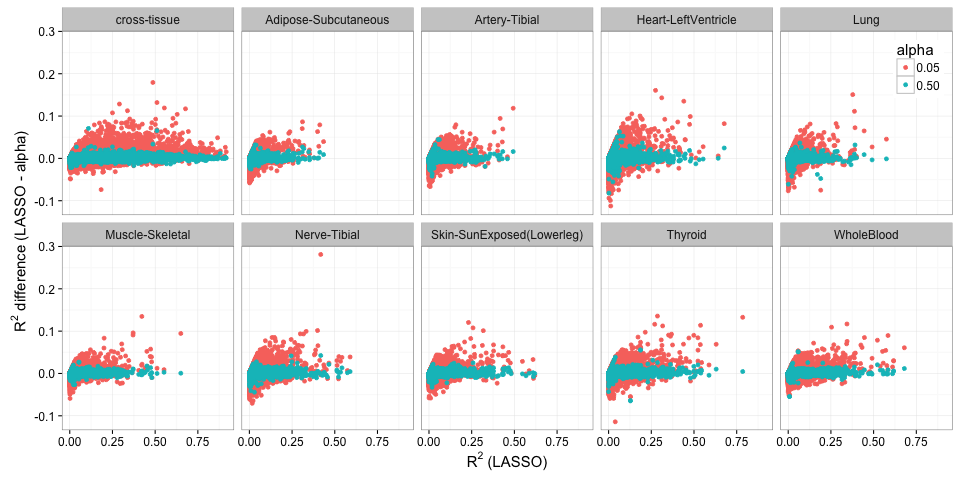

Supplement: S11 Fig — The difference between the cross validated R2 of the LASSO model and the elastic net model mixing parameters 0.05 and 0.5 for autosomal protein coding genes per cross-tissue and tissue-specific gene expression traits. Elastic net with α = 0.5 values hover around zero, meaning that it has similar predictive performance to LASSO. The R2 difference of the more polygenic model (elastic net with α = 0.05) is mostly above the 0 line, indicating that this model performs worse than the LASSO model across decomposed tissues. (PNG) [file pgen.1006423.s011.png]

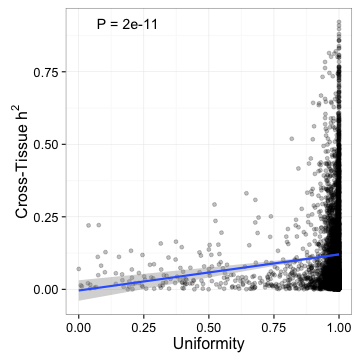

Supplement: S12 Fig — The measure of uniformity was computed using the posterior probability of a gene being actively regulated in a tissue, PPA, from the Flutre et al. [33] multi-tissue eQTL analysis. Genes with PPA concentrated in one tissue were assigned small values of the uniformity measure whereas genes with PPA uniformly distributed across tissues were assigned high value of uniformity measure. See Methods for the entropy-based definition of uniformity. The blue line is the best fit linear regression and shows a significant positive correlation between h2 and uniformity (β = 0.12, P = 2e − 11). (PNG) [file pgen.1006423.s012.png]
