# Supplementary material for: Survey of the Heritability and Sparse Architecture of Gene Expression Traits across Human Tissues
Source: PLoS Genet. 2016 Nov 11;12(11):e1006423. doi: 10.1371/journal.pgen.1006423 (PMC5106030; doi:10.1371/journal.pgen.1006423)
Supplement: S1 Table — Expression levels derived by Orthogonal Tissue Decomposition and h2 estimated using the --reml-no-constrain method. (PDF) [file pgen.1006423.s013.pdf]

S1 Table. Estimates of cross-tissue and tissue-specific local h^2. Expression levels derived by Orthogonal Tissue Decomposition and h^2 estimated using the `--reml-no-constrain` method.

tissue	n	mean h^2	% FDR<0.1	num FDR<0.1	num expressed
Cross-tissue	450	0.062	20.2	2995	14861
Adipose - Subcutaneous	298	0.017	9.5	1408	14861
Adrenal Gland	126	0.037	8.9	1329	14861
Artery - Aorta	198	0.022	9.8	1449	14861
Artery - Coronary	119	0.048	8.1	1211	14861
Artery - Tibial	285	0.022	8.5	1262	14861
Brain - Anterior cingulate cortex (BA24)	72	0.037	10.9	1627	14861
Brain - Caudate (basal ganglia)	100	0.042	9.3	1388	14861
Brain - Cerebellar Hemisphere	89	0.046	10.9	1627	14861
Brain - Cerebellum	103	0.041	10.9	1618	14861
Brain - Cortex	96	0.053	10.0	1480	14861
Brain - Frontal Cortex (BA9)	92	0.037	10.6	1569	14861
Brain - Hippocampus	81	0.033	10.9	1618	14861
Brain - Hypothalamus	81	0.020	12.4	1839	14861
Brain - Nucleus accumbens (basal ganglia)	93	0.040	10.7	1585	14861
Brain - Putamen (basal ganglia)	82	0.033	10.6	1581	14861
Breast - Mammary Tissue	183	0.020	9.2	1367	14861
Cells - EBV-transformed lymphocytes	115	0.045	8.9	1323	14861
Cells - Transformed fibroblasts	272	0.019	9.0	1334	14861
Colon - Sigmoid	124	0.024	10.6	1578	14861
Colon - Transverse	170	0.022	10.6	1573	14861
Esophagus - Gastroesophageal Junction	127	0.029	9.1	1358	14861
Esophagus - Mucosa	242	0.026	8.2	1220	14861
Esophagus - Muscularis	219	0.024	9.0	1344	14861
Heart - Atrial Appendage	159	0.031	9.7	1437	14861
Heart - Left Ventricle	190	0.025	8.9	1325	14861
Liver	98	0.036	10.2	1519	14861
Lung	279	0.018	8.8	1308	14861
Muscle - Skeletal	361	0.020	9.0	1342	14861
Nerve - Tibial	256	0.026	8.3	1235	14861
Ovary	85	0.043	10.2	1523	14861
Pancreas	150	0.036	9.4	1396	14861
Pituitary	87	0.044	10.0	1481	14861
Skin - Not Sun Exposed (Suprapubic)	196	0.041	6.3	938	14861
Skin - Sun Exposed (Lower leg)	303	0.027	6.9	1020	14861
Small Intestine - Terminal Ileum	77	0.046	11.7	1733	14861
Spleen	89	0.062	9.7	1448	14861
Stomach	171	0.020	10.3	1532	14861
Testis	157	0.042	10.5	1561	14861
Thyroid	279	0.024	8.7	1293	14861
Whole Blood	339	0.025	7.7	1150	14861

All tissues are from the GTEx Project. Mean heritability (h^2) is calculated across genes for each tissue. The percentage (%) and number (num) of genes with significant h^2 estimates (FDR < 0.1) in each tissue are reported.
